# Diseases of the Blood

**Published:** 1906-02-10

**Authors:** 


### DISEASES OF THE BLOOD.

**Blood-Pressure.**—Rolleston [1] acknowledges that we have little reason to think that arterial disease leads to high blood-pressure, though the reverse is common enough. When he tries to analyse the causes of high pressure, he finds that many of them are uncertain. Excess of food, and especially of the purin bodies, toxins produced by faulty digestion, or metabolism, or by past diseases such as typhoid, failure of excretion and nerve influences, have all been credited with producing it, while of late, deficiency of the thyroid or over-activity of the supra-renals have been added to the list. Indeed, in granular kidney with high tension a hyperplasia of the supra-renals has been observed in some instances, but Rolleston shows that this cannot be the cause of the high pressure, for the increase only occurs in the cortex which does not produce adrenalin. Such an increase, if it has any effect at all on the tension, probably implies an effort to antagonise the existing poisons, and to lower the pressure. Arteriosclerosis indeed, as Rolleston points out, may lead to granular kidney and uræmia, and thus high pressure may be fatal from uræmia, cerebral hæmorrhage, or heart-failure, as well as in other ways. As to the means of treatment, moderation in drink and food, especially as to proteids, is important, and exercise without excessive effort. Iodides possibly stimulate the thyroid, which in turn is supposed to antagonise the supra-renals. The nitrites have hitherto been disappointing and probably require to be used in much larger doses to produce any marked result. Loomis recommends five grains of chloral hydrate every four hours, but great care must be taken with drugs which, like it or aconite, act directly on the heart. Finally, salines and mercury reduce tension by the removal of various poisons through the intestines. G. Oliver[2] has given us another instalment of the results of his researches on pressure measurements. He shows that while the Riva Rocci instrument gives the maximum or systolic pressure, Leonard Hills, records the diastolic pressure. He has himself devised a hæmomanometer consisting of a horizontal tube which ends in a closed air bulb, and contains a short column of coloured absolute alcohol as an indicator. In other respects it resembles an ordinary sphygmometer, but it is more accurate, since there is practically no inertia in the indicator, and from its sensitiveness the diastolic pressure can be measured in the finger tips as well as in the limbs. The instrument, too, is more portable and less liable to accidents than the ordinary ones. The practical objections to taking the pressure in the arm on account of the discomfort caused to the patient, has led him to examine measurements taken from the forearm with the band arranged spirally, and he finds that there is no difference in readings taken from the two places. He also insists that reliable readings are given by his hæmodynamometer without any of the discomforts caused by these manometers, though it must be confessed that it gives only the diastolic pressure. In answering the question how far does the pressure vary in different parts of the circulatory system, he finds that there is no appreciable alteration between the shoulder and the wrist, but that it falls by more than half between the wrist and the tip of the finger. When the circulation is stimulated by exercise or digestion, the diastolic pressure rises in the most distal area, and when this amounts to 30 per cent., the pressure in the larger arteries falls and may even become the same as in the most distal ones. Thus pressure of 100 in the arm and 45 at the third phalanx, may be replaced by 85 everywhere. The effect of exercise and other vasodilators then upon the peripheral vessels is to raise the pressure, while in the large vessels there is at first a rise both of systolic and diastolic pressures, but eventually both fall below normal, the diastolic first. If we select a period, such as one hour before meals, when the circulation is at rest, we shall find that the pressure in a given individual is remarkably regular from day to day—namely, 95 to 100 diastolic pressure, and 115 to 120 systolic in healthy young men. The higher the tension the more easily is the pressure varied by digestion or exercise. If we ask what is the meaning of systolic and diastolic pressures, the answer seems to be that the systolic represents the force of the ventricular contraction, while the diastolic gives us a measure of the peripheral resistance, which is at least as important for us to know as the force of ventricular contraction. It is interesting to note that in the treatment of disease most drugs have been found so inefficient that movement cures, such as the Nauheim treatment, have been largely used instead. Such exercises mean, of course, the opening up of a large additional area for the blood. Oliver has found that certain drugs of the aromatic series have a remarkable effect in regulating pressure, and thinks that a really active agent of this kind may probably be discovered ere long.

**Coagulability.**—It will be remembered that A. E. Wright showed long ago that the coagulation power of the blood could in many cases be increased by the administration of calcium chloride. Further experiments by Wright and Paramore[3] recently have shown that the full effect is produced within an hour, but that individuals differ in their power of absorbing calcium. The lactate of calcium was also tried, and found to be pleasanter to take and even more efficacious than the chloride. The full effects of a dose of one drachm were produced in three-quarters of an hour, and lasted for some days, but it is not clear that all persons can absorb even the lactate. In the case of a hæmophiliac patient increased coagulability was only obtained when the salt was given hypodermically. It may be added here that the lactate alone should be used for hypodermic injections, and that its solution should be of less than 1 in 20 strength. Another coagulating agent was found in magnesium carbonate, which has a similar power of quickening the time in which clotting takes place. This explains its common therapeutic employment in urticarias due to the ingestion of decalcifying agents such as soap enemas, rhubarb, gooseberries, and other sour fruits and wines, though the result is obtained not by neutralising a supposed acid, but by the addition of a substance which favours coagulation. Both calcium and magnesium salts then have the property of raising the coagulation power of the blood in persons who can absorb them, and are therefore useful in hæmorrhages and serous exudations such as urticaria. In persons placed on a milk diet, the effect of the large amount of these salts necessarily ingested may lead to actual thrombosis. The writers have also shown that coagulability may be reduced by giving citric acid, though there seems at present a difficulty in keeping it down to a low level permanently. In maintaining a permanent high degree of coagulability by calcium salts it is desirable to test the state of the blood frequently and to reduce the dose or stop the drug when the maximum effect is produced.

[1] Clin. Jour., June 21. [2] Lancet, July 22. [3] Ibid., Oct. 14.

